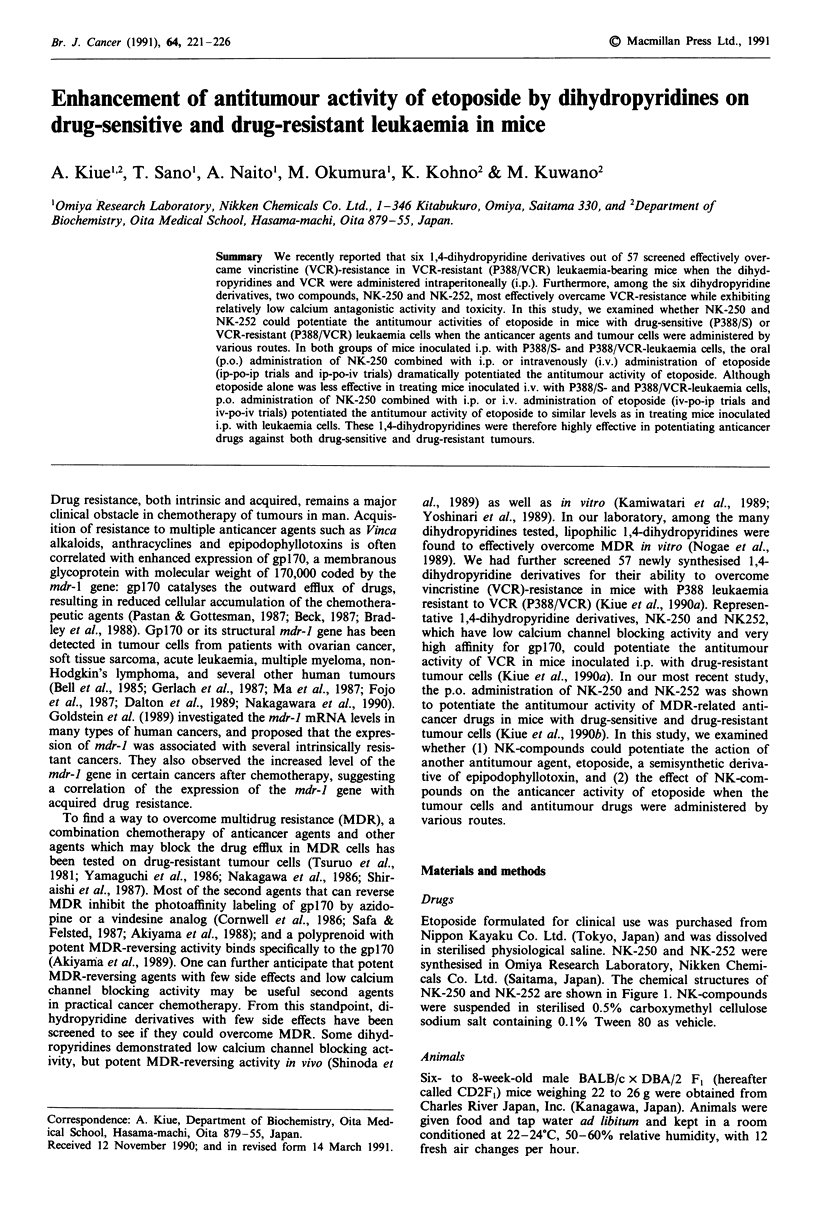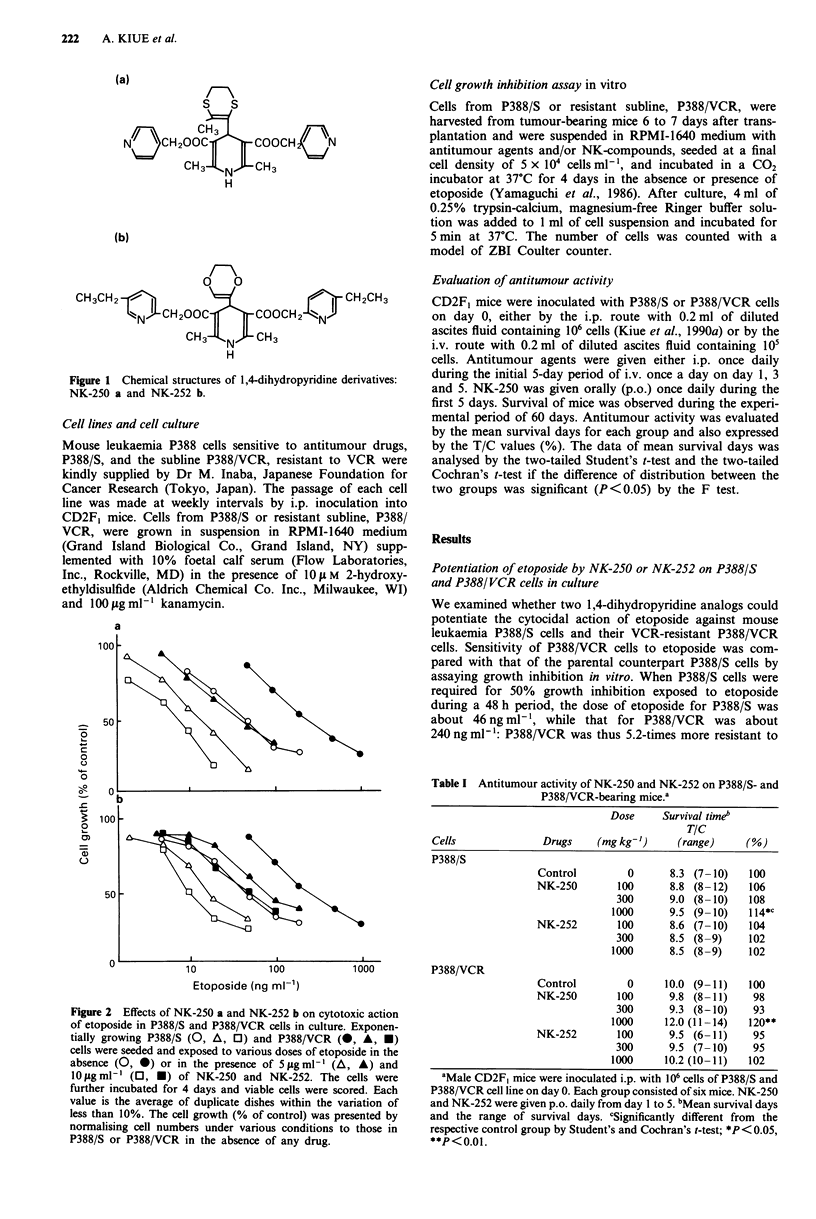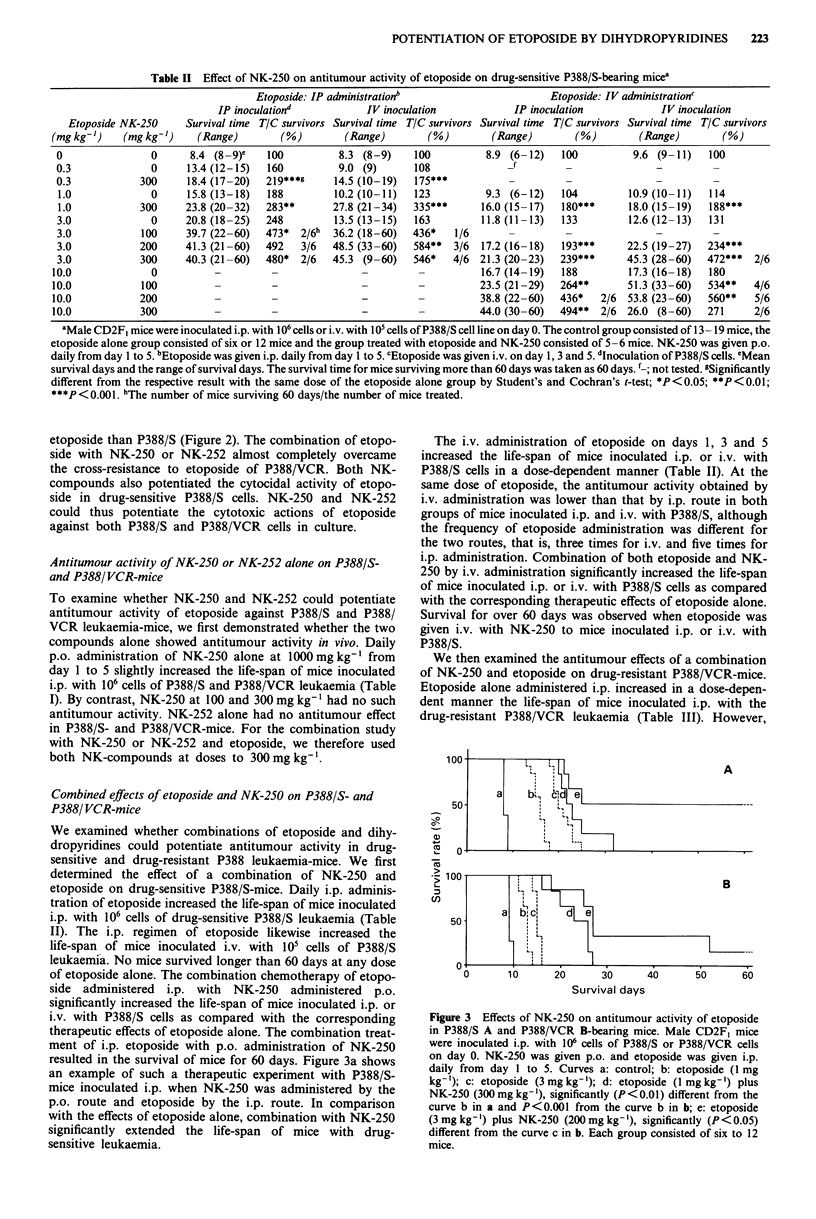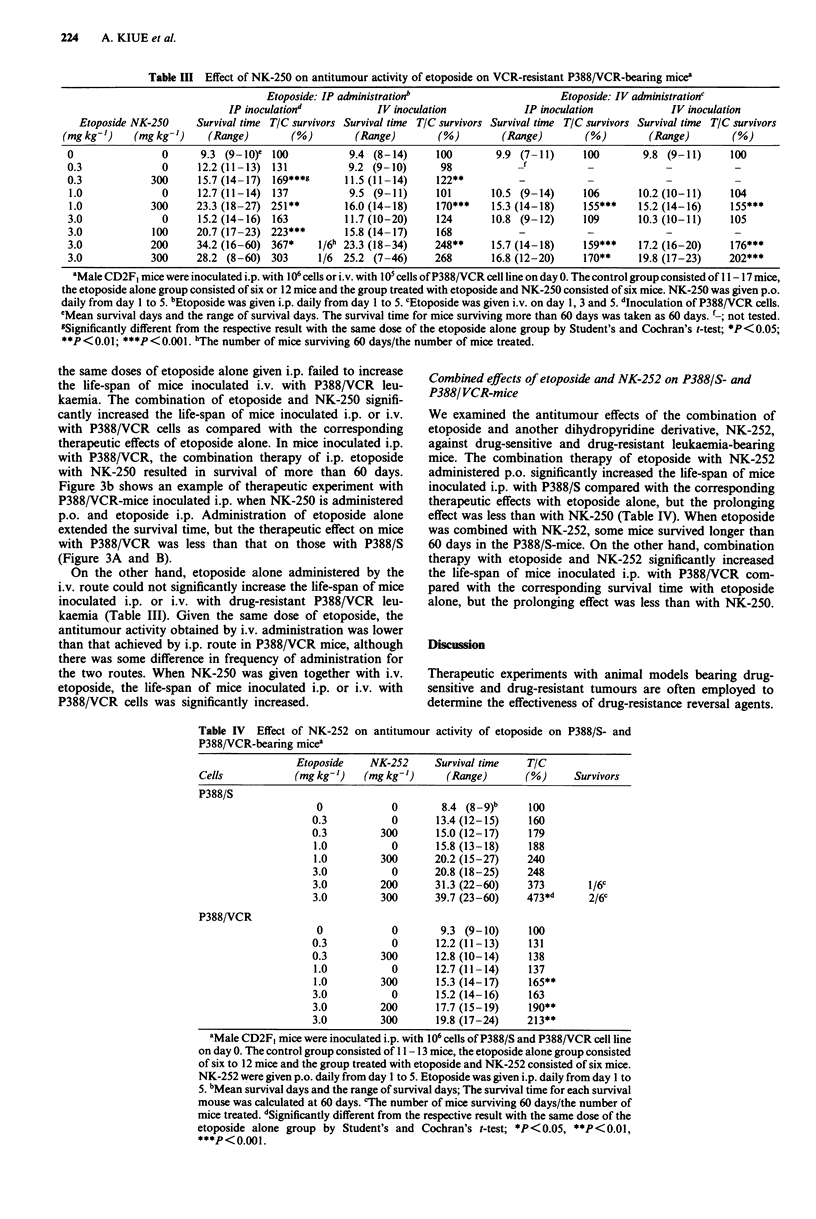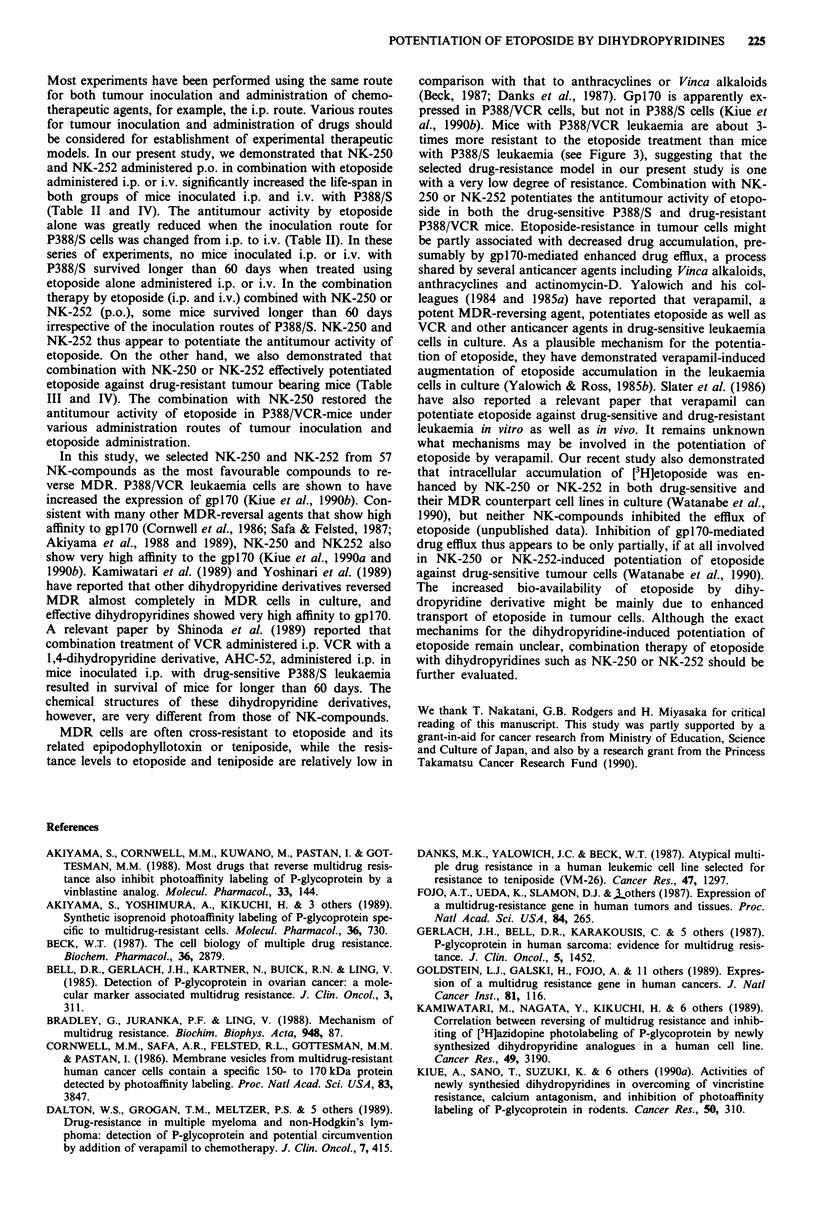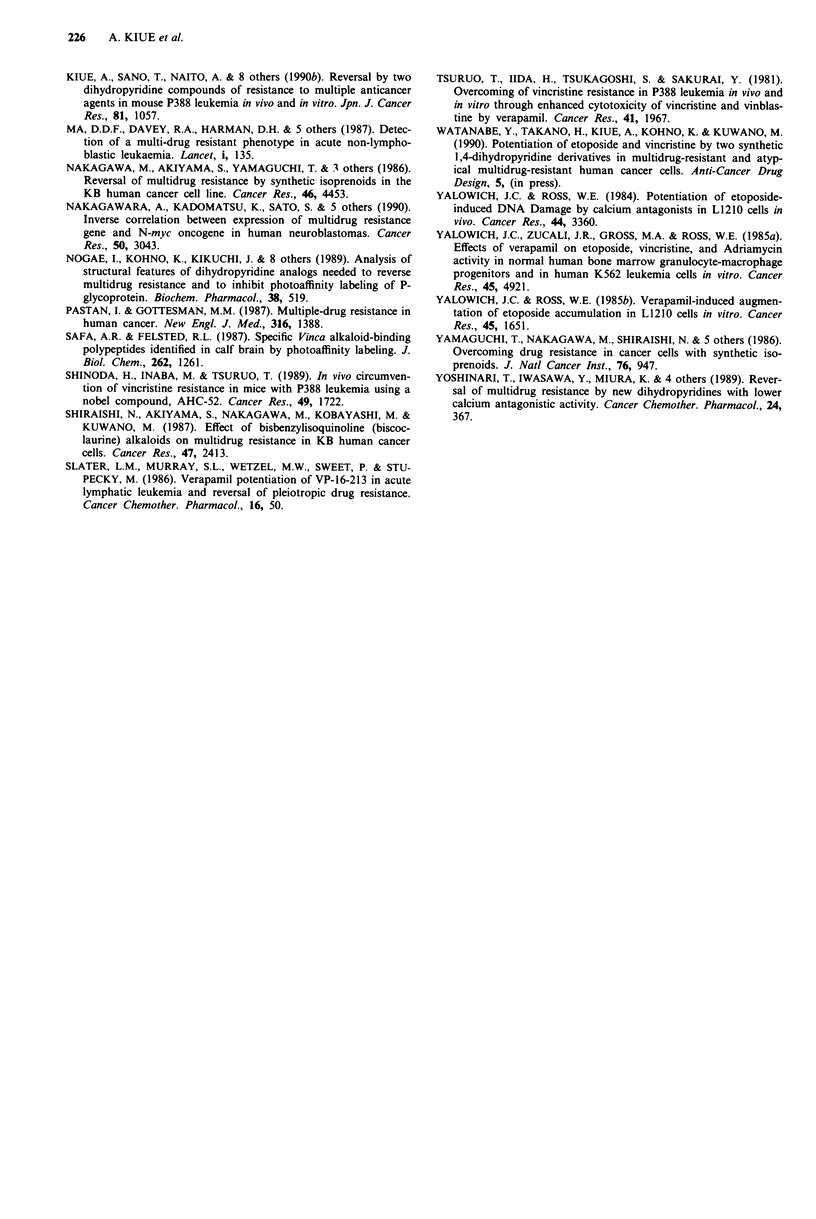# Enhancement of antitumour activity of etoposide by dihydropyridines on drug-sensitive and drug-resistant leukaemia in mice.

**DOI:** 10.1038/bjc.1991.280

**Published:** 1991-08

**Authors:** A. Kiue, T. Sano, A. Naito, M. Okumura, K. Kohno, M. Kuwano

**Affiliations:** Omiya Research Laboratory, Nikken Chemicals Co. Ltd., Saitama, Japan.

## Abstract

We recently reported that six 1,4-dihydropyridine derivatives out of 57 screened effectively over-came vincristine (VCR)-resistance in VCR-resistant (P388/VCR) leukaemia-bearing mice when the dihydropyridines and VCR were administered intraperitoneally (i.p.). Furthermore, among the six dihydropyridine derivatives, two compounds, NK-250 and NK-252, most effectively overcame VCR-resistance while exhibiting relatively low calcium antagonistic activity and toxicity. In this study, we examined whether NK-250 and NK-252 could potentiate the antitumour activities of etoposide in mice with drug-sensitive (P388/S) or VCR-resistant (P388/VCR) leukaemia cells when the anticancer agents and tumour cells were administered by various routes. In both groups of mice inoculated i.p. with P388/S- and P388/VCR-leukaemia cells, the oral (p.o.) administration of NK-250 combined with i.p. or intravenously (i.v.) administration of etoposide (ip-po-ip trials and ip-po-iv trials) dramatically potentiated the antitumour activity of etoposide. Although etoposide alone was less effective in treating mice inoculated i.v. with P388/S- and P388/VCR-leukaemia cells, p.o. administration of NK-250 combined with i.p. or i.v. administration of etoposide (iv-po-ip trials and iv-po-iv trials) potentiated the antitumour activity of etoposide to similar levels as in treating mice inoculated i.p. with leukaemia cells. These 1,4-dihydropyridines were therefore highly effective in potentiating anticancer drugs against both drug-sensitive and drug-resistant tumours.


Br. J. Cancer (1991), 64, 221–226 © Macmillan Press Ltd., 1991

### Enhancement of antitumour activity of etoposide by dihydropyridines on drug-sensitive and drug-resistant leukaemia in mice

A. Kiue[1,2], T. Sano[1], A. Naito[1], M. Okumura[1], K. Kohno[2] & M. Kuwano[2]

[1]*Omiya Research Laboratory, Nikken Chemicals Co. Ltd., 1-346 Kitabukuro, Omiya, Saitama 330, and* [2]*Department of Biochemistry, Oita Medical School, Hasama-machi, Oita 879-55, Japan.*

**Summary** We recently reported that six 1,4-dihydropyridine derivatives out of 57 screened effectively overcame vincristine (VCR)-resistance in VCR-resistant (P388/VCR) leukaemia-bearing mice when the dihydropyridines and VCR were administered intraperitoneally (i.p.). Furthermore, among the six dihydropyridine derivatives, two compounds, NK-250 and NK-252, most effectively overcame VCR-resistance while exhibiting relatively low calcium antagonistic activity and toxicity. In this study, we examined whether NK-250 and NK-252 could potentiate the antitumour activities of etoposide in mice with drug-sensitive (P388/S) or VCR-resistant (P388/VCR) leukaemia cells when the anticancer agents and tumour cells were administered by various routes. In both groups of mice inoculated i.p. with P388/S- and P388/VCR-leukaemia cells, the oral (p.o.) administration of NK-250 combined with i.p. or intravenously (i.v.) administration of etoposide (ip-po-ip trials and ip-po-iv trials) dramatically potentiated the antitumour activity of etoposide. Although etoposide alone was less effective in treating mice inoculated i.v. with P388/S- and P388/VCR-leukaemia cells, p.o. administration of NK-250 combined with i.p. or i.v. administration of etoposide (iv-po-ip trials and iv-po-iv trials) potentiated the antitumour activity of etoposide to similar levels as in treating mice inoculated i.p. with leukaemia cells. These 1,4-dihydropyridines were therefore highly effective in potentiating anticancer drugs against both drug-sensitive and drug-resistant tumours.

Drug resistance, both intrinsic and acquired, remains a major clinical obstacle in chemotherapy of tumours in man. Acquisition of resistance to multiple anticancer agents such as *Vinca* alkaloids, anthracyclines and epipodophyllotoxins is often correlated with enhanced expression of gp170, a membranous glycoprotein with molecular weight of 170,000 coded by the *mdr*-1 gene: gp170 catalyses the outward efflux of drugs, resulting in reduced cellular accumulation of the chemotherapeutic agents (Pastan & Gottesman, 1987; Beck, 1987; Bradley *et al.*, 1988). Gp170 or its structural *mdr-1* gene has been detected in tumour cells from patients with ovarian cancer, soft tissue sarcoma, acute leukaemia, multiple myeloma, non-Hodgkin's lymphoma, and several other human tumours (Bell *et al.*, 1985; Gerlach *et al.*, 1987; Ma *et al.*, 1987; Fojo *et al.*, 1987; Dalton *et al.*, 1989; Nakagawara *et al.*, 1990). Goldstein *et al.* (1989) investigated the *mdr-1* mRNA levels in many types of human cancers, and proposed that the expression of *mdr-1* was associated with several intrinsically resistant cancers. They also observed the increased level of the *mdr-1* gene in certain cancers after chemotherapy, suggesting a correlation of the expression of the *mdr-1* gene with acquired drug resistance.

To find a way to overcome multidrug resistance (MDR), a combination chemotherapy of anticancer agents and other agents which may block the drug efflux in MDR cells has been tested on drug-resistant tumour cells (Tsuruo *et al.*, 1981; Yamaguchi *et al.*, 1986; Nakagawa *et al.*, 1986; Shiraishi *et al.*, 1987). Most of the second agents that can reverse MDR inhibit the photoaffinity labeling of gp170 by azidopine or a vindesine analog (Cornwell *et al.*, 1986; Safa & Felsted, 1987; Akiyama *et al.*, 1988); and a polyprenoid with potent MDR-reversing activity binds specifically to the gp170 (Akiyama *et al.*, 1989). One can further anticipate that potent MDR-reversing agents with few side effects and low calcium channel blocking activity may be useful second agents in practical cancer chemotherapy. From this standpoint, dihydropyridine derivatives with few side effects have been screened to see if they could overcome MDR. Some dihydropyridines demonstrated low calcium channel blocking activity, but potent MDR-reversing activity *in vivo* (Shinoda *et al.*, 1989) as well as *in vitro* (Kamiwatari *et al.*, 1989; Yoshinari *et al.*, 1989). In our laboratory, among the many dihydropyridines tested, lipophilic 1,4-dihydropyridines were found to effectively overcome MDR *in vitro* (Nogae *et al.*, 1989). We had further screened 57 newly synthesised 1,4-dihydropyridine derivatives for their ability to overcome vincristine (VCR)-resistance in mice with P388 leukaemia resistant to VCR (P388/VCR) (Kiue *et al.*, 1990*a*). Representative 1,4-dihydropyridine derivatives, NK-250 and NK252, which have low calcium channel blocking activity and very high affinity for gp170, could potentiate the antitumour activity of VCR in mice inoculated i.p. with drug-resistant tumour cells (Kiue *et al.*, 1990*a*). In our most recent study, the p.o. administration of NK-250 and NK-252 was shown to potentiate the antitumour activity of MDR-related anticancer drugs in mice with drug-sensitive and drug-resistant tumour cells (Kiue *et al.*, 1990*b*). In this study, we examined whether (1) NK-compounds could potentiate the action of another antitumour agent, etoposide, a semisynthetic derivative of epipodophyllotoxin, and (2) the effect of NK-compounds on the anticancer activity of etoposide when the tumour cells and antitumour drugs were administered by various routes.

### Materials and methods

### *Drugs*

Etoposide formulated for clinical use was purchased from Nippon Kayaku Co. Ltd. (Tokyo, Japan) and was dissolved in sterilised physiological saline. NK-250 and NK-252 were synthesised in Omiya Research Laboratory, Nikken Chemicals Co. Ltd. (Saitama, Japan). The chemical structures of NK-250 and NK-252 are shown in Figure 1. NK-compounds were suspended in sterilised 0.5% carboxymethyl cellulose sodium salt containing 0.1% Tween 80 as vehicle.

### *Animals*

Six- to 8-week-old male BALB/c × DBA/2 $F_1$ (hereafter called $CD2F_1$) mice weighing 22 to 26 g were obtained from Charles River Japan, Inc. (Kanagawa, Japan). Animals were given food and tap water *ad libitum* and kept in a room conditioned at 22–24°C, 50–60% relative humidity, with 12 fresh air changes per hour.

Correspondence: A. Kiue, Department of Biochemistry, Oita Medical School, Hasama-machi, Oita 879-55, Japan.
Received 12 November 1990; and in revised form 14 March 1991.

**Figure 1** Chemical structures of 1,4-dihydropyridine derivatives: NK-250 **a** and NK-252 **b**.

*Cell lines and cell culture*

Mouse leukaemia P388 cells sensitive to antitumour drugs, P388/S, and the subline P388/VCR, resistant to VCR were kindly supplied by Dr M. Inaba, Japanese Foundation for Cancer Research (Tokyo, Japan). The passage of each cell line was made at weekly intervals by i.p. inoculation into $CD2F_1$ mice. Cells from P388/S or resistant subline, P388/VCR, were grown in suspension in RPMI-1640 medium (Grand Island Biological Co., Grand Island, NY) supplemented with 10% foetal calf serum (Flow Laboratories, Inc., Rockville, MD) in the presence of 10 µM 2-hydroxyethyldisulfide (Aldrich Chemical Co. Inc., Milwaukee, WI) and 100 $\mu g\ ml^{-1}$ kanamycin.

**Figure 2** Effects of NK-250 **a** and NK-252 **b** on cytotoxic action of etoposide in P388/S and P388/VCR cells in culture. Exponentially growing P388/S (○, △, □) and P388/VCR (●, ▲, ■) cells were seeded and exposed to various doses of etoposide in the absence (○, ●) or in the presence of 5 $\mu g\ ml^{-1}$ (△, ▲) and 10 $\mu g\ ml^{-1}$ (□, ■) of NK-250 and NK-252. The cells were further incubated for 4 days and viable cells were scored. Each value is the average of duplicate dishes within the variation of less than 10%. The cell growth (% of control) was presented by normalising cell numbers under various conditions to those in P388/S or P388/VCR in the absence of any drug.

*Cell growth inhibition assay* in vitro

Cells from P388/S or resistant subline, P388/VCR, were harvested from tumour-bearing mice 6 to 7 days after transplantation and were suspended in RPMI-1640 medium with antitumour agents and/or NK-compounds, seeded at a final cell density of $5 \times 10^4$ cells $ml^{-1}$, and incubated in a $CO_2$ incubator at 37°C for 4 days in the absence or presence of etoposide (Yamaguchi *et al.*, 1986). After culture, 4 ml of 0.25% trypsin-calcium, magnesium-free Ringer buffer solution was added to 1 ml of cell suspension and incubated for 5 min at 37°C. The number of cells was counted with a model of ZBI Coulter counter.

*Evaluation of antitumour activity*

$CD2F_1$ mice were inoculated with P388/S or P388/VCR cells on day 0, either by the i.p. route with 0.2 ml of diluted ascites fluid containing $10^6$ cells (Kiue *et al.*, 1990*a*) or by the i.v. route with 0.2 ml of diluted ascites fluid containing $10^5$ cells. Antitumour agents were given either i.p. once daily during the initial 5-day period of i.v. once a day on day 1, 3 and 5. NK-250 was given orally (p.o.) once daily during the first 5 days. Survival of mice was observed during the experimental period of 60 days. Antitumour activity was evaluated by the mean survival days for each group and also expressed by the T/C values (%). The data of mean survival days was analysed by the two-tailed Student's *t*-test and the two-tailed Cochran's *t*-test if the difference of distribution between the two groups was significant ($P<0.05$) by the F test.

### Results

*Potentiation of etoposide by NK-250 or NK-252 on P388/S and P388/VCR cells in culture*

We examined whether two 1,4-dihydropyridine analogs could potentiate the cytocidal action of etoposide against mouse leukaemia P388/S cells and their VCR-resistant P388/VCR cells. Sensitivity of P388/VCR cells to etoposide was compared with that of the parental counterpart P388/S cells by assaying growth inhibition *in vitro*. When P388/S cells were required for 50% growth inhibition exposed to etoposide during a 48 h period, the dose of etoposide for P388/S was about 46 ng $ml^{-1}$, while that for P388/VCR was about 240 ng $ml^{-1}$: P388/VCR was thus 5.2-times more resistant to

**Table I** Antitumour activity of NK-250 and NK-252 on P388/S- and P388/VCR-bearing mice.[a]

| *Cells* | *Drugs* | *Dose* (*mg kg*$^{-1}$) | *Survival time*[b] (*range*) | *T/C* (*%*) |
|---|---|---|---|---|
| P388/S | | | | |
| | Control | 0 | 8.3 (7–10) | 100 |
| | NK-250 | 100 | 8.8 (8–12) | 106 |
| | | 300 | 9.0 (8–10) | 108 |
| | | 1000 | 9.5 (9–10) | 114*[c] |
| | NK-252 | 100 | 8.6 (7–10) | 104 |
| | | 300 | 8.5 (8–9) | 102 |
| | | 1000 | 8.5 (8–9) | 102 |
| P388/VCR | | | | |
| | Control | 0 | 10.0 (9–11) | 100 |
| | NK-250 | 100 | 9.8 (8–11) | 98 |
| | | 300 | 9.3 (8–10) | 93 |
| | | 1000 | 12.0 (11–14) | 120** |
| | NK-252 | 100 | 9.5 (6–11) | 95 |
| | | 300 | 9.5 (7–10) | 95 |
| | | 1000 | 10.2 (10–11) | 102 |

[a]Male $CD2F_1$ mice were inoculated i.p. with $10^6$ cells of P388/S and P388/VCR cell line on day 0. Each group consisted of six mice. NK-250 and NK-252 were given p.o. daily from day 1 to 5. [b]Mean survival days and the range of survival days. [c]Significantly different from the respective control group by Student's and Cochran's *t*-test; *$P<0.05$, **$P<0.01$.

**Table II** Effect of NK-250 on antitumour activity of etoposide on drug-sensitive P388/S-bearing mice[a]

| | | Etoposide: IP administration[b] | | | | Etoposide: IV administration[c] | | | |
|---|---|---|---|---|---|---|---|---|---|
| | | IP inoculation[d] | | IV inoculation | | IP inoculation | | IV inoculation | |
| Etoposide ($mg\ kg^{-1}$) | NK-250 ($mg\ kg^{-1}$) | Survival time (Range) | T/C survivors (%) | Survival time (Range) | T/C survivors (%) | Survival time (Range) | T/C survivors (%) | Survival time (Range) | T/C survivors (%) |
| 0 | 0 | 8.4 (8–9)[e] | 100 | 8.3 (8–9) | 100 | 8.9 (6–12) | 100 | 9.6 (9–11) | 100 |
| 0.3 | 0 | 13.4 (12–15) | 160 | 9.0 (9) | 108 | –[f] | – | – | – |
| 0.3 | 300 | 18.4 (17–20) | 219***[g] | 14.5 (10–19) | 175*** | – | – | – | – |
| 1.0 | 0 | 15.8 (13–18) | 188 | 10.2 (10–11) | 123 | 9.3 (6–12) | 104 | 10.9 (10–11) | 114 |
| 1.0 | 300 | 23.8 (20–32) | 283** | 27.8 (21–34) | 335*** | 16.0 (15–17) | 180*** | 18.0 (15–19) | 188*** |
| 3.0 | 0 | 20.8 (18–25) | 248 | 13.5 (13–15) | 163 | 11.8 (11–13) | 133 | 12.6 (12–13) | 131 |
| 3.0 | 100 | 39.7 (22–60) | 473* 2/6[h] | 36.2 (18–60) | 436* 1/6 | – | – | – | – |
| 3.0 | 200 | 41.3 (21–60) | 492 3/6 | 48.5 (33–60) | 584** 3/6 | 17.2 (16–18) | 193*** | 22.5 (19–27) | 234*** |
| 3.0 | 300 | 40.3 (21–60) | 480* 2/6 | 45.3 (9–60) | 546* 4/6 | 21.3 (20–23) | 239*** | 45.3 (28–60) | 472*** 2/6 |
| 10.0 | 0 | – | – | – | – | 16.7 (14–19) | 188 | 17.3 (16–18) | 180 |
| 10.0 | 100 | – | – | – | – | 23.5 (21–29) | 264** | 51.3 (33–60) | 534** 4/6 |
| 10.0 | 200 | – | – | – | – | 38.8 (22–60) | 436* 2/6 | 53.8 (23–60) | 560** 5/6 |
| 10.0 | 300 | – | – | – | – | 44.0 (30–60) | 494** 2/6 | 26.0 (8–60) | 271 2/6 |

[a]Male $CD2F_1$ mice were inoculated i.p. with $10^6$ cells or i.v. with $10^5$ cells of P388/S cell line on day 0. The control group consisted of 13–19 mice, the etoposide alone group consisted of six or 12 mice and the group treated with etoposide and NK-250 consisted of 5–6 mice. NK-250 was given p.o. daily from day 1 to 5. [b]Etoposide was given i.p. daily from day 1 to 5. [c]Etoposide was given i.v. on day 1, 3 and 5. [d]Inoculation of P388/S cells. [e]Mean survival days and the range of survival days. The survival time for mice surviving more than 60 days was taken as 60 days. [f]–; not tested. [g]Significantly different from the respective result with the same dose of the etoposide alone group by Student's and Cochran's *t*-test; $*P<0.05$; $**P<0.01$; $***P<0.001$. [h]The number of mice surviving 60 days/the number of mice treated.

etoposide than P388/S (Figure 2). The combination of etoposide with NK-250 or NK-252 almost completely overcame the cross-resistance to etoposide of P388/VCR. Both NK-compounds also potentiated the cytocidal activity of etoposide in drug-sensitive P388/S cells. NK-250 and NK-252 could thus potentiate the cytotoxic actions of etoposide against both P388/S and P388/VCR cells in culture.

### *Antitumour activity of NK-250 or NK-252 alone on P388/S- and P388/VCR-mice*

To examine whether NK-250 and NK-252 could potentiate antitumour activity of etoposide against P388/S and P388/VCR leukaemia-mice, we first demonstrated whether the two compounds alone showed antitumour activity *in vivo*. Daily p.o. administration of NK-250 alone at $1000\ mg\ kg^{-1}$ from day 1 to 5 slightly increased the life-span of mice inoculated i.p. with $10^6$ cells of P388/S and P388/VCR leukaemia (Table I). By contrast, NK-250 at 100 and $300\ mg\ kg^{-1}$ had no such antitumour activity. NK-252 alone had no antitumour effect in P388/S- and P388/VCR-mice. For the combination study with NK-250 or NK-252 and etoposide, we therefore used both NK-compounds at doses to $300\ mg\ kg^{-1}$.

### *Combined effects of etoposide and NK-250 on P388/S- and P388/VCR-mice*

We examined whether combinations of etoposide and dihydropyridines could potentiate antitumour activity in drug-sensitive and drug-resistant P388 leukaemia-mice. We first determined the effect of a combination of NK-250 and etoposide on drug-sensitive P388/S-mice. Daily i.p. administration of etoposide increased the life-span of mice inoculated i.p. with $10^6$ cells of drug-sensitive P388/S leukaemia (Table II). The i.p. regimen of etoposide likewise increased the life-span of mice inoculated i.v. with $10^5$ cells of P388/S leukaemia. No mice survived longer than 60 days at any dose of etoposide alone. The combination chemotherapy of etoposide administered i.p. with NK-250 administered p.o. significantly increased the life-span of mice inoculated i.p. or i.v. with P388/S cells as compared with the corresponding therapeutic effects of etoposide alone. The combination treatment of i.p. etoposide with p.o. administration of NK-250 resulted in the survival of mice for 60 days. Figure 3a shows an example of such a therapeutic experiment with P388/S-mice inoculated i.p. when NK-250 was administered by the p.o. route and etoposide by the i.p. route. In comparison with the effects of etoposide alone, combination with NK-250 significantly extended the life-span of mice with drug-sensitive leukaemia.

The i.v. administration of etoposide on days 1, 3 and 5 increased the life-span of mice inoculated i.p. or i.v. with P388/S cells in a dose-dependent manner (Table II). At the same dose of etoposide, the antitumour activity obtained by i.v. administration was lower than that by i.p. route in both groups of mice inoculated i.p. and i.v. with P388/S, although the frequency of etoposide administration was different for the two routes, that is, three times for i.v. and five times for i.p. administration. Combination of both etoposide and NK-250 by i.v. administration significantly increased the life-span of mice inoculated i.p. or i.v. with P388/S cells as compared with the corresponding therapeutic effects of etoposide alone. Survival for over 60 days was observed when etoposide was given i.v. with NK-250 to mice inoculated i.p. or i.v. with P388/S.

We then examined the antitumour effects of a combination of NK-250 and etoposide on drug-resistant P388/VCR-mice. Etoposide alone administered i.p. increased in a dose-dependent manner the life-span of mice inoculated i.p. with the drug-resistant P388/VCR leukaemia (Table III). However,

**Figure 3** Effects of NK-250 on antitumour activity of etoposide in P388/S **A** and P388/VCR **B**-bearing mice. Male $CD2F_1$ mice were inoculated i.p. with $10^6$ cells of P388/S or P388/VCR cells on day 0. NK-250 was given p.o. and etoposide was given i.p. daily from day 1 to 5. Curves a: control; b: etoposide ($1\ mg\ kg^{-1}$); c: etoposide ($3\ mg\ kg^{-1}$); d: etoposide ($1\ mg\ kg^{-1}$) plus NK-250 ($300\ mg\ kg^{-1}$), significantly ($P<0.01$) different from the curve b in **a** and $P<0.001$ from the curve b in **b**; e: etoposide ($3\ mg\ kg^{-1}$) plus NK-250 ($200\ mg\ kg^{-1}$), significantly ($P<0.05$) different from the curve c in **b**. Each group consisted of six to 12 mice.

**Table III** Effect of NK-250 on antitumour activity of etoposide on VCR-resistant P388/VCR-bearing mice[a]

| | | Etoposide: IP administration[b] | | | | Etoposide: IV administration[c] | | | |
|---|---|---|---|---|---|---|---|---|---|
| | | IP inoculation[d] | | IV inoculation | | IP inoculation | | IV inoculation | |
| Etoposide (mg kg$^{-1}$) | NK-250 (mg kg$^{-1}$) | Survival time (Range) | T/C survivors (%) | Survival time (Range) | T/C survivors (%) | Survival time (Range) | T/C survivors (%) | Survival time (Range) | T/C survivors (%) |
| 0 | 0 | 9.3 (9–10)[e] | 100 | 9.4 (8–14) | 100 | 9.9 (7–11) | 100 | 9.8 (9–11) | 100 |
| 0.3 | 0 | 12.2 (11–13) | 131 | 9.2 (9–10) | 98 | –[f] | – | – | – |
| 0.3 | 300 | 15.7 (14–17) | 169***[g] | 11.5 (11–14) | 122** | – | – | – | – |
| 1.0 | 0 | 12.7 (11–14) | 137 | 9.5 (9–11) | 101 | 10.5 (9–14) | 106 | 10.2 (10–11) | 104 |
| 1.0 | 300 | 23.3 (18–27) | 251** | 16.0 (14–18) | 170*** | 15.3 (14–18) | 155*** | 15.2 (14–16) | 155*** |
| 3.0 | 0 | 15.2 (14–16) | 163 | 11.7 (10–20) | 124 | 10.8 (9–12) | 109 | 10.3 (10–11) | 105 |
| 3.0 | 100 | 20.7 (17–23) | 223*** | 15.8 (14–17) | 168 | – | – | – | – |
| 3.0 | 200 | 34.2 (16–60) | 367* 1/6[h] | 23.3 (18–34) | 248** | 15.7 (14–18) | 159*** | 17.2 (16–20) | 176*** |
| 3.0 | 300 | 28.2 (8–60) | 303 1/6 | 25.2 (7–46) | 268 | 16.8 (12–20) | 170** | 19.8 (17–23) | 202*** |

[a]Male CD2F$_1$ mice were inoculated i.p. with $10^6$ cells or i.v. with $10^5$ cells of P388/VCR cell line on day 0. The control group consisted of 11–17 mice, the etoposide alone group consisted of six or 12 mice and the group treated with etoposide and NK-250 consisted of six mice. NK-250 was given p.o. daily from day 1 to 5. [b]Etoposide was given i.p. daily from day 1 to 5. [c]Etoposide was given i.v. on day 1, 3 and 5. [d]Inoculation of P388/VCR cells. [e]Mean survival days and the range of survival days. The survival time for mice surviving more than 60 days was taken as 60 days. [f]–; not tested. [g]Significantly different from the respective result with the same dose of the etoposide alone group by Student's and Cochran's $t$-test; *$P<0.05$; **$P<0.01$; ***$P<0.001$. [h]The number of mice surviving 60 days/the number of mice treated.

the same doses of etoposide alone given i.p. failed to increase the life-span of mice inoculated i.v. with P388/VCR leukaemia. The combination of etoposide and NK-250 significantly increased the life-span of mice inoculated i.p. or i.v. with P388/VCR cells as compared with the corresponding therapeutic effects of etoposide alone. In mice inoculated i.p. with P388/VCR, the combination therapy of i.p. etoposide with NK-250 resulted in survival of more than 60 days. Figure 3b shows an example of therapeutic experiment with P388/VCR-mice inoculated i.p. when NK-250 is administered p.o. and etoposide i.p. Administration of etoposide alone extended the survival time, but the therapeutic effect on mice with P388/VCR was less than that on those with P388/S (Figure 3A and B).

On the other hand, etoposide alone administered by the i.v. route could not significantly increase the life-span of mice inoculated i.p. or i.v. with drug-resistant P388/VCR leukaemia (Table III). Given the same dose of etoposide, the antitumour activity obtained by i.v. administration was lower than that achieved by i.p. route in P388/VCR mice, although there was some difference in frequency of administration for the two routes. When NK-250 was given together with i.v. etoposide, the life-span of mice inoculated i.p. or i.v. with P388/VCR cells was significantly increased.

### *Combined effects of etoposide and NK-252 on P388/S- and P388/VCR-mice*

We examined the antitumour effects of the combination of etoposide and another dihydropyridine derivative, NK-252, against drug-sensitive and drug-resistant leukaemia-bearing mice. The combination therapy of etoposide with NK-252 administered p.o. significantly increased the life-span of mice inoculated i.p. with P388/S compared with the corresponding therapeutic effects with etoposide alone, but the prolonging effect was less than with NK-250 (Table IV). When etoposide was combined with NK-252, some mice survived longer than 60 days in the P388/S-mice. On the other hand, combination therapy with etoposide and NK-252 significantly increased the life-span of mice inoculated i.p. with P388/VCR compared with the corresponding survival time with etoposide alone, but the prolonging effect was less than with NK-250.

### Discussion

Therapeutic experiments with animal models bearing drug-sensitive and drug-resistant tumours are often employed to determine the effectiveness of drug-resistance reversal agents.

**Table IV** Effect of NK-252 on antitumour activity of etoposide on P388/S- and P388/VCR-bearing mice[a]

| Cells | Etoposide (mg kg$^{-1}$) | NK-252 (mg kg$^{-1}$) | Survival time (Range) | T/C (%) | Survivors |
|---|---|---|---|---|---|
| P388/S | | | | | |
| | 0 | 0 | 8.4 (8–9)[b] | 100 | |
| | 0.3 | 0 | 13.4 (12–15) | 160 | |
| | 0.3 | 300 | 15.0 (12–17) | 179 | |
| | 1.0 | 0 | 15.8 (13–18) | 188 | |
| | 1.0 | 300 | 20.2 (15–27) | 240 | |
| | 3.0 | 0 | 20.8 (18–25) | 248 | |
| | 3.0 | 200 | 31.3 (22–60) | 373 | 1/6[c] |
| | 3.0 | 300 | 39.7 (23–60) | 473*[d] | 2/6[c] |
| P388/VCR | | | | | |
| | 0 | 0 | 9.3 (9–10) | 100 | |
| | 0.3 | 0 | 12.2 (11–13) | 131 | |
| | 0.3 | 300 | 12.8 (10–14) | 138 | |
| | 1.0 | 0 | 12.7 (11–14) | 137 | |
| | 1.0 | 300 | 15.3 (14–17) | 165** | |
| | 3.0 | 0 | 15.2 (14–16) | 163 | |
| | 3.0 | 200 | 17.7 (15–19) | 190** | |
| | 3.0 | 300 | 19.8 (17–24) | 213** | |

[a]Male CD2F$_1$ mice were inoculated i.p. with $10^6$ cells of P388/S and P388/VCR cell line on day 0. The control group consisted of 11–13 mice, the etoposide alone group consisted of six to 12 mice and the group treated with etoposide and NK-252 consisted of six mice. NK-252 were given p.o. daily from day 1 to 5. Etoposide was given i.p. daily from day 1 to 5. [b]Mean survival days and the range of survival days; The survival time for each survival mouse was calculated at 60 days. [c]The number of mice surviving 60 days/the number of mice treated. [d]Significantly different from the respective result with the same dose of the etoposide alone group by Student's and Cochran's $t$-test; *$P<0.05$, **$P<0.01$, ***$P<0.001$.

Most experiments have been performed using the same route for both tumour inoculation and administration of chemotherapeutic agents, for example, the i.p. route. Various routes for tumour inoculation and administration of drugs should be considered for establishment of experimental therapeutic models. In our present study, we demonstrated that NK-250 and NK-252 administered p.o. in combination with etoposide administered i.p. or i.v. significantly increased the life-span in both groups of mice inoculated i.p. and i.v. with P388/S (Table II and IV). The antitumour activity by etoposide alone was greatly reduced when the inoculation route for P388/S cells was changed from i.p. to i.v. (Table II). In these series of experiments, no mice inoculated i.p. or i.v. with P388/S survived longer than 60 days when treated using etoposide alone administered i.p. or i.v. In the combination therapy by etoposide (i.p. and i.v.) combined with NK-250 or NK-252 (p.o.), some mice survived longer than 60 days irrespective of the inoculation routes of P388/S. NK-250 and NK-252 thus appear to potentiate the antitumour activity of etoposide. On the other hand, we also demonstrated that combination with NK-250 or NK-252 effectively potentiated etoposide against drug-resistant tumour bearing mice (Table III and IV). The combination with NK-250 restored the antitumour activity of etoposide in P388/VCR-mice under various administration routes of tumour inoculation and etoposide administration.

In this study, we selected NK-250 and NK-252 from 57 NK-compounds as the most favourable compounds to reverse MDR. P388/VCR leukaemia cells are shown to have increased the expression of gp170 (Kiue *et al*., 1990*b*). Consistent with many other MDR-reversal agents that show high affinity to gp170 (Cornwell *et al*., 1986; Safa & Felsted, 1987; Akiyama *et al*., 1988 and 1989), NK-250 and NK252 also show very high affinity to the gp170 (Kiue *et al*., 1990*a* and 1990*b*). Kamiwatari *et al*. (1989) and Yoshinari *et al*. (1989) have reported that other dihydropyridine derivatives reversed MDR almost completely in MDR cells in culture, and effective dihydropyridines showed very high affinity to gp170. A relevant paper by Shinoda *et al*. (1989) reported that combination treatment of VCR administered i.p. VCR with a 1,4-dihydropyridine derivative, AHC-52, administered i.p. in mice inoculated i.p. with drug-sensitive P388/S leukaemia resulted in survival of mice for longer than 60 days. The chemical structures of these dihydropyridine derivatives, however, are very different from those of NK-compounds.

MDR cells are often cross-resistant to etoposide and its related epipodophyllotoxin or teniposide, while the resistance levels to etoposide and teniposide are relatively low in comparison with that to anthracyclines or *Vinca* alkaloids (Beck, 1987; Danks *et al*., 1987). Gp170 is apparently expressed in P388/VCR cells, but not in P388/S cells (Kiue *et al*., 1990*b*). Mice with P388/VCR leukaemia are about 3-times more resistant to the etoposide treatment than mice with P388/S leukaemia (see Figure 3), suggesting that the selected drug-resistance model in our present study is one with a very low degree of resistance. Combination with NK-250 or NK-252 potentiates the antitumour activity of etoposide in both the drug-sensitive P388/S and drug-resistant P388/VCR mice. Etoposide-resistance in tumour cells might be partly associated with decreased drug accumulation, presumably by gp170-mediated enhanced drug efflux, a process shared by several anticancer agents including *Vinca* alkaloids, anthracyclines and actinomycin-D. Yalowich and his colleagues (1984 and 1985*a*) have reported that verapamil, a potent MDR-reversing agent, potentiates etoposide as well as VCR and other anticancer agents in drug-sensitive leukaemia cells in culture. As a plausible mechanism for the potentiation of etoposide, they have demonstrated verapamil-induced augmentation of etoposide accumulation in the leukaemia cells in culture (Yalowich & Ross, 1985*b*). Slater *et al*. (1986) have also reported a relevant paper that verapamil can potentiate etoposide against drug-sensitive and drug-resistant leukaemia *in vitro* as well as *in vivo*. It remains unknown what mechanisms may be involved in the potentiation of etoposide by verapamil. Our recent study also demonstrated that intracellular accumulation of [$^3$H]etoposide was enhanced by NK-250 or NK-252 in both drug-sensitive and their MDR counterpart cell lines in culture (Watanabe *et al*., 1990), but neither NK-compounds inhibited the efflux of etoposide (unpublished data). Inhibition of gp170-mediated drug efflux thus appears to be only partially, if at all involved in NK-250 or NK-252-induced potentiation of etoposide against drug-sensitive tumour cells (Watanabe *et al*., 1990). The increased bio-availability of etoposide by dihydropyridine derivative might be mainly due to enhanced transport of etoposide in tumour cells. Although the exact mechanims for the dihydropyridine-induced potentiation of etoposide remain unclear, combination therapy of etoposide with dihydropyridines such as NK-250 or NK-252 should be further evaluated.

We thank T. Nakatani, G.B. Rodgers and H. Miyasaka for critical reading of this manuscript. This study was partly supported by a grant-in-aid for cancer research from Ministry of Education, Science and Culture of Japan, and also by a research grant from the Princess Takamatsu Cancer Research Fund (1990).

### References

AKIYAMA, S., CORNWELL, M.M., KUWANO, M., PASTAN, I. & GOTTESMAN, M.M. (1988). Most drugs that reverse multidrug resistance also inhibit photoaffinity labeling of P-glycoprotein by a vinblastine analog. *Molecul. Pharmacol.*, **33**, 144.

AKIYAMA, S., YOSHIMURA, A., KIKUCHI, H. & 3 others (1989). Synthetic isoprenoid photoaffinity labeling of P-glycoprotein specific to multidrug-resistant cells. *Molecul. Pharmacol.*, **36**, 730.

BECK, W.T. (1987). The cell biology of multiple drug resistance. *Biochem. Pharmacol.*, **36**, 2879.

BELL, D.R., GERLACH, J.H., KARTNER, N., BUICK, R.N. & LING, V. (1985). Detection of P-glycoprotein in ovarian cancer: a molecular marker associated multidrug resistance. *J. Clin. Oncol.*, **3**, 311.

BRADLEY, G., JURANKA, P.F. & LING, V. (1988). Mechanism of multidrug resistance. *Biochim. Biophys. Acta*, **948**, 87.

CORNWELL, M.M., SAFA, A.R., FELSTED, R.L., GOTTESMAN, M.M. & PASTAN, I. (1986). Membrane vesicles from multidrug-resistant human cancer cells contain a specific 150- to 170 kDa protein detected by photoaffinity labeling. *Proc. Natl Acad. Sci. USA*, **83**, 3847.

DALTON, W.S., GROGAN, T.M., MELTZER, P.S. & 5 others (1989). Drug-resistance in multiple myeloma and non-Hodgkin's lymphoma: detection of P-glycoprotein and potential circumvention by addition of verapamil to chemotherapy. *J. Clin. Oncol.*, **7**, 415.

DANKS, M.K., YALOWICH, J.C. & BECK, W.T. (1987). Atypical multiple drug resistance in a human leukemic cell line selected for resistance to teniposide (VM-26). *Cancer Res.*, **47**, 1297.

FOJO, A.T., UEDA, K., SLAMON, D.J. & 3 others (1987). Expression of a multidrug-resistance gene in human tumors and tissues. *Proc. Natl Acad. Sci. USA*, **84**, 265.

GERLACH, J.H., BELL, D.R., KARAKOUSIS, C. & 5 others (1987). P-glycoprotein in human sarcoma: evidence for multidrug resistance. *J. Clin. Oncol.*, **5**, 1452.

GOLDSTEIN, L.J., GALSKI, H., FOJO, A. & 11 others (1989). Expression of a multidrug resistance gene in human cancers. *J. Natl Cancer Inst.*, **81**, 116.

KAMIWATARI, M., NAGATA, Y., KIKUCHI, H. & 6 others (1989). Correlation between reversing of multidrug resistance and inhibiting of [$^3$H]azidopine photolabeling of P-glycoprotein by newly synthesized dihydropyridine analogues in a human cell line. *Cancer Res.*, **49**, 3190.

KIUE, A., SANO, T., SUZUKI, K. & 6 others (1990*a*). Activities of newly synthesied dihydropyridines in overcoming of vincristine resistance, calcium antagonism, and inhibition of photoaffinity labeling of P-glycoprotein in rodents. *Cancer Res.*, **50**, 310.

KIUE, A., SANO, T., NAITO, A. & 8 others (1990*b*). Reversal by two dihydropyridine compounds of resistance to multiple anticancer agents in mouse P388 leukemia *in vivo* and *in vitro*. *Jpn. J. Cancer Res.*, **81**, 1057.

MA, D.D.F., DAVEY, R.A., HARMAN, D.H. & 5 others (1987). Detection of a multi-drug resistant phenotype in acute non-lymphoblastic leukaemia. *Lancet*, **i**, 135.

NAKAGAWA, M., AKIYAMA, S., YAMAGUCHI, T. & 3 others (1986). Reversal of multidrug resistance by synthetic isoprenoids in the KB human cancer cell line. *Cancer Res.*, **46**, 4453.

NAKAGAWARA, A., KADOMATSU, K., SATO, S. & 5 others (1990). Inverse correlation between expression of multidrug resistance gene and N-*myc* oncogene in human neuroblastomas. *Cancer Res.*, **50**, 3043.

NOGAE, I., KOHNO, K., KIKUCHI, J. & 8 others (1989). Analysis of structural features of dihydropyridine analogs needed to reverse multidrug resistance and to inhibit photoaffinity labeling of P-glycoprotein. *Biochem. Pharmacol.*, **38**, 519.

PASTAN, I. & GOTTESMAN, M.M. (1987). Multiple-drug resistance in human cancer. *New Engl. J. Med.*, **316**, 1388.

SAFA, A.R. & FELSTED, R.L. (1987). Specific *Vinca* alkaloid-binding polypeptides identified in calf brain by photoaffinity labeling. *J. Biol. Chem.*, **262**, 1261.

SHINODA, H., INABA, M. & TSURUO, T. (1989). *In vivo* circumvention of vincristine resistance in mice with P388 leukemia using a nobel compound, AHC-52. *Cancer Res.*, **49**, 1722.

SHIRAISHI, N., AKIYAMA, S., NAKAGAWA, M., KOBAYASHI, M. & KUWANO, M. (1987). Effect of bisbenzylisoquinoline (biscoclaurine) alkaloids on multidrug resistance in KB human cancer cells. *Cancer Res.*, **47**, 2413.

SLATER, L.M., MURRAY, S.L., WETZEL, M.W., SWEET, P. & STUPECKY, M. (1986). Verapamil potentiation of VP-16-213 in acute lymphatic leukemia and reversal of pleiotropic drug resistance. *Cancer Chemother. Pharmacol.*, **16**, 50.

TSURUO, T., IIDA, H., TSUKAGOSHI, S. & SAKURAI, Y. (1981). Overcoming of vincristine resistance in P388 leukemia *in vivo* and *in vitro* through enhanced cytotoxicity of vincristine and vinblastine by verapamil. *Cancer Res.*, **41**, 1967.

WATANABE, Y., TAKANO, H., KIUE, A., KOHNO, K. & KUWANO, M. (1990). Potentiation of etoposide and vincristine by two synthetic 1,4-dihydropyridine derivatives in multidrug-resistant and atypical multidrug-resistant human cancer cells. *Anti-Cancer Drug Design*, **5**, (in press).

YALOWICH, J.C. & ROSS, W.E. (1984). Potentiation of etoposide-induced DNA Damage by calcium antagonists in L1210 cells *in vivo*. *Cancer Res.*, **44**, 3360.

YALOWICH, J.C., ZUCALI, J.R., GROSS, M.A. & ROSS, W.E. (1985*a*). Effects of verapamil on etoposide, vincristine, and Adriamycin activity in normal human bone marrow granulocyte-macrophage progenitors and in human K562 leukemia cells *in vitro*. *Cancer Res.*, **45**, 4921.

YALOWICH, J.C. & ROSS, W.E. (1985*b*). Verapamil-induced augmentation of etoposide accumulation in L1210 cells *in vitro*. *Cancer Res.*, **45**, 1651.

YAMAGUCHI, T., NAKAGAWA, M., SHIRAISHI, N. & 5 others (1986). Overcoming drug resistance in cancer cells with synthetic isoprenoids. *J. Natl Cancer Inst.*, **76**, 947.

YOSHINARI, T., IWASAWA, Y., MIURA, K. & 4 others (1989). Reversal of multidrug resistance by new dihydropyridines with lower calcium antagonistic activity. *Cancer Chemother. Pharmacol.*, **24**, 367.